# Integrating Machine Learning into Free Energy Perturbation Workflows

**DOI:** 10.1021/acs.jcim.5c01449

**Published:** 2025-09-17

**Authors:** Donald J. M. van Pinxteren, Willem Jespers

**Affiliations:** Department of Medicinal Chemistry, Photopharmacology and Imaging, Groningen Research Institute of Pharmacy (GRIP), University of Groningen, Antonius Deusinglaan 1, 9713 AV Groningen, The Netherlands

## Abstract

Free energy perturbation (FEP) methods are among the most accurate tools in structure-based drug design for predicting protein–ligand binding affinities. However, their adoption remains limited due to high computational demands and complex setup procedures. This review explores how integrating machine learning (ML), especially active learning (AL) and deep learning (DL), can enhance the efficiency, accessibility, accuracy, and precision of FEP workflows. It examines three key areas where ML has been successfully applied: sampling strategies, protocol optimization, and force field development. AL algorithms can significantly reduce the number of FEP calculations needed during virtual screening by guiding the molecule selection. DL-based protein–ligand cofolding methods such as AlphaFold, NeuralPLexer, and DragonFold enable the automated generation of accurate complex structures for FEP, bypassing traditional docking and preparation steps. Additionally, ML-derived neural network potentials (NNPs), trained on quantum mechanical data, offer improved force field accuracy, although at the cost of higher computational expenses. This review emphasizes a hybrid approach combining human expertise with ML tools as the most promising strategy for accelerating and democratizing FEP-based drug discovery. Future developments in this interdisciplinary space are expected to expand the scope and impact of computer-aided drug design across pharmaceutical and materials science applications.

## Graphical abstract

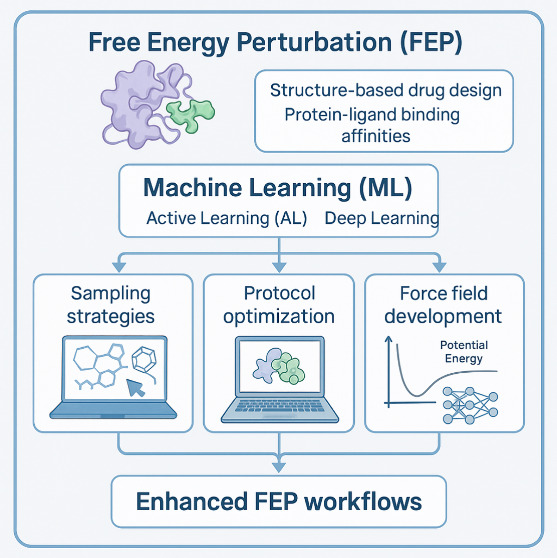

## 1 Introduction

In the field of drug discovery and optimization, computational methods have been evolving rapidly. Due to increasing computational capacity, open-source chemical databases and protein structures, and improvements in efficiency, reliability, and accuracy in methods, computer-aided drug discovery (CADD) has become an important tool in drug discovery and optimization.^1,2^ CADD includes ligand-based drug design (LBDD), where compounds are typically screened based on chemical similarity to the structures of known ligands, and structure-based drug design (SBDD), where a new drug is designed based on the structure of a protein. The latter includes methods such as molecular docking, molecular dynamics (MD) simulations, and free energy perturbation (FEP) methods. With the introduction and constant improvement of machine learning (ML)-based protein structure prediction models like AlphaFold, more high-resolution protein structures are becoming available, and SBDD is fully adopted as a ligand discovery and optimization method in the traditional drug discovery process.^3,4^

While molecular docking and MD simulations can give great insights into the interactions ligands have with a protein’s binding site, FEP methods can be used to predict binding affinities quantitatively. In this review, we will focus on two applications of FEP, namely, absolute free energy perturbation (ABFE) and relative free energy perturbation (RBFE). Each of these methods has in common that they scale the potential energies of two states through a series of small steps, popularly referred to as alchemical changes. During each step, MD simulations are used to calculate the change in free energy by using, for instance, FEP, thermodynamic integration (TI), or the (multistate) Bennet acceptance ratio ((M)­BAR).^5^ By summing these energy differences across all steps, FEP yields the overall binding free energy difference between two states in a thermodynamic cycle. In RBFE, these include two ligands in a solvent and protein leg; for ABFE, these are the original ligand and its noninteracting dummy atoms using the same legs.

RBFE is the most commonly used method in drug discovery campaigns. It is used in lead optimization processes and is less expensive and more accurate than ABFE.^6,7^ However, RBFE requires that the alchemical changes between the two ligands are minor, and a reference ligand is needed to calculate the actual binding affinity. This potentially can be overcome using ABFE, although modeling the relevant (pseudo) apo state of the protein is a key challenge.^7^ In drug discovery campaigns, residue FEP is commonly used to calculate the effect of protein mutations to ligand binding or alternatively to predict changes in affinity of a peptide to a protein.^8^

Since the systematic investigation of the performance of FEP in retrospective analyses, the prospective application of FEP methods has been reported in various cases.^1^ For instance, Bayer utilized FEP to develop inhibitors targeting the Kirsten rat sarcoma (KRAS) gene with the G12C mutation, enabling the identification of a new scaffold with a novel binding mode that enhanced affinity.^9^ Schrödinger applied FEP potency predictors to guide the design of a tyrosine kinase 2 (TYK2) inhibitor with improved binding characteristics.^10^ Janssen leveraged FEP to discover new [1,2,4]­triazolo­[1,5-*a*]­pyrimidine phosphodiesterase 2A (PDE2A) inhibitors, including compounds with unexpectedly high activity.^11^ Similarly, AstraZeneca employed FEP in the lead optimization of an ATP-competitive c-MET inhibitor.^12^ Additionally, two studies by Tandaric et al. and Majellaro et al. used QligFEP and QresFEP to elucidate the binding mode of the adenosine A_2B_ receptor (A_2B_AR).^13,14^

FEP methods have demonstrated significant value and accuracy in the drug discovery and optimization process. However, they are still hindered by challenges such as high computational demands and the significant effort required for system preparation. Thus, developing more efficient methods that require less computational power and data input could greatly enhance the ability to screen larger molecular libraries and explore the chemical space more comprehensively. This advancement would accelerate early-stage drug discovery and potentially lower the cost and time associated with identifying promising drug candidates. Such improvements could also make these tools more accessible to research groups with limited computational resources.

Machine learning (ML) algorithms, particularly deep learning (DL) and active learning (AL), hold significant potential to enhance the efficiency and accuracy of data-driven processes across various scientific domains.^15,16^ Their integration into FEP methodologies presents a promising opportunity to improve both the reliability and the computational performance of these techniques. Recent studies have demonstrated the application of ML in optimizing force fields, automating FEP protocol development, and improving sampling strategies. Furthermore, a comprehensive review by Qian et al. provides a detailed overview of recent advances in alchemical transformation methods, and a paper by Mey et al. elaborates on the best practices for alchemical free energy calculations allowing a systematic application of FEP.^5,17^ In this review, we aim to provide further insights into how the convergence of ML and FEP may further empower CADD within both academic and industrial pharmaceutical research.

## 2 Machine Learning Implementations in Free Energy Perturbation Methods

This section addresses three critical components of FEP workflows that are being enhanced through the integration of ML: sampling strategies, protocol setup, and force field development. These areas of advancement aim to reduce the number of FEP calculations required for comprehensive compound library screening, improve the accuracy and robustness of FEP setup procedures, and increase the accuracy of FEP and underlying MD simulations. Each subsection provides an in-depth exploration of the relevant methodologies and ML-driven innovations that contribute to more efficient and reliable FEP computations, summarized in Table 1.

**Table 1:** Overview of the Three Sections of the FEP Workflow Discussed in This Review and the Use of ML Therein

**subsection**	**section of the FEP workflow**	**references**
2.1 AL for FEP Sample Selection	screening efficiency	^18,19,20,21,22^
2.2 FEP Protocol and Protein Setup	FEP preparation	^3,4,29,30,31,32,33,34,35,36,37,38,39,40^
2.3 Force Field Enhancements	MD accuracy and efficiency	^47,48,49,50,51,52,53,54,55,57,59,60,61^

### 2.1 AL for FEP Sample Selection

AL has emerged as a promising strategy for improving sampling efficiency in the screening of chemical libraries using FEP. Although still relatively underexplored, several studies have demonstrated the application of AL frameworks in which quantitative structure–activity relationship (QSAR) models are trained on FEP-generated data to prioritize the selection of new candidate molecules. The primary objective of this approach is to maximize the identification of high-affinity ligands while minimizing the number of costly FEP simulations required.

Scheme 1 shows a graphical overview of the general AL framework. A chemical library of interest is selected. This set is iteratively split into subsets: a training set for the ML model, a subset for the FEP calculations, and an independent test set. After FEP calculations are performed on the subset, the results are used to retrain the QSAR model, and its performance is assessed on the test set. In the next iteration, a new subset of the test set is acquired for FEP calculations to add to the training set for the QSAR model. Generally, two types of selection criteria can be used. An exploitative or greedy acquisition focuses on the compounds most likely to have the highest binding affinity. Alternatively, an explorative acquisition focuses on compounds that have the highest uncertainty in the predicted binding affinity from the machine learning model.

**Scheme 1:**
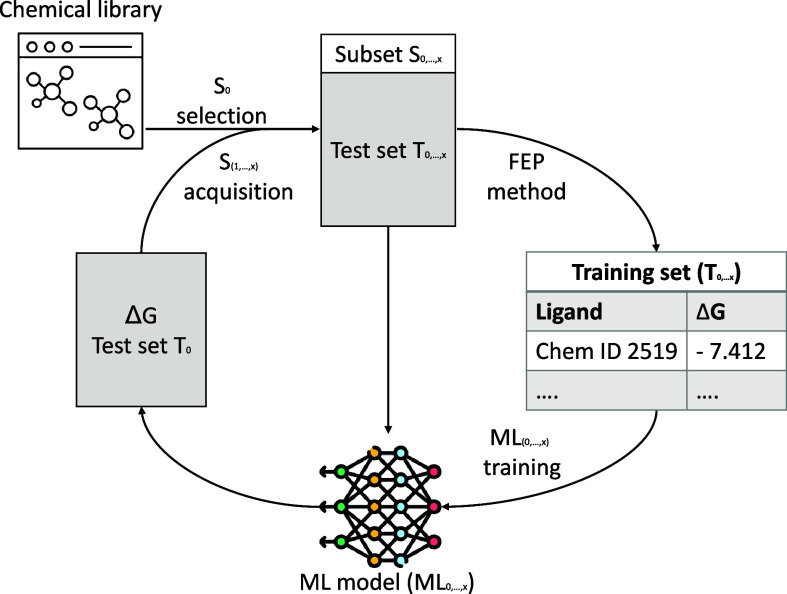
Schematic Overview of Active Learning Enhanced Sampling Techniques for Library Screening with FEP

A typically used metric to assess the performance of an AL-driven screen is the recall, which is defined as the number of high-affinity compounds found divided by the total number of high-affinity compounds present in the data set. By focusing on this metric, the model does not miss potent candidates while spending resources on the most informative or promising molecules. Key parameters investigated in AL-FEP workflows include the choice of ML algorithm for QSAR modeling, molecular descriptors used as input features, initial training set composition, batch size, acquisition function, and the number of iterative selection rounds, which we discuss in more detail in the next paragraphs.^18–22^

To assess the efficiency of AL-FEP in exploring the chemical space, Khalak et al. tested and optimized multiple molecular descriptors and acquisition methods. Three types of descriptors were tested: RDKit-generated molecular fingerprints; MedusaNet descriptors representing physics-based descriptors; and protein–ligand interaction fingerprints, named PLEC.^18,23–25^ In addition, Khalak et al. tested the acquisition method in the AL loop: *random selection* (selecting random compounds at each iteration); *greedy selection* (choosing the top predicted binders at each iteration); *uncertainty selection* (picking compounds with the highest uncertainty in the binding affinity prediction); *mixed strategy* (first selecting the top predicted binders for three iterations and then the most uncertain); and a *narrowing strategy* (combining broad selection in the first three iterations with a subsequent switch to a greedy approach). During the initial iterations, several models were trained, each using different sets of the ligand descriptors described earlier, and the five models with the lowest cross-validation root-mean-square error (RMSE) were identified. From each of these models, the 20 best predicted binders were then selected. Regarding the acquisition method, overall, uncertain, and random ligand selection sampling broadly covered the chemical library and offered a better overall description of the chemical space. In the case of the molecular descriptors, RDKit’s molecular fingerprints outperformed the interaction fingerprints as well as the physics-based descriptors. However, to efficiently identify the most potent binders, other strategies, such as a greedy or narrowing approach, were recommended by the authors.^18^

Thompson et al. tested different ML algorithms, acquisition methods, initial subset selection methods, and batch sizes of 20, 40, 60, 80, or 100 molecules per iteration. In an effort to systematically assess the effect of several optimization strategies for AL, Thompson et al. created a data set of 10,000 RBFE calculations on a congeneric series.^19^ The biggest impact in performance came from different batch sizes per iteration. Almost no difference in performance was measured between the initial subset selection methods and ML algorithm choice. They observed a slight improvement in performance with greedy acquisition methods. More importantly, they found that using batch sizes of 60 or more molecules per iteration was sufficient to identify at least 50% of the top-binding compounds within five iterations (sampling a total of 300 molecules). In contrast, smaller batch sizes required more iterations to achieve the same level of high-affinity compound identification across all active learning setup configurations. For example, 10 iterations with a batch size of 20, sampling a total of 200 compounds, would recall around 40% of the 100 highest affinity compounds with the same AL setups. As conclusion, they sampled 5% of a 10,000 data set identifying 50% of the top molecules.^19^ Notably, Thompson et al. state in the discussion that the AL-FEP method configuration depends on the data set and target the experiment is performed on. For this data set, they concluded that a simple random initial subset with a greedy acquisition function performs as well as complex methods trying to balance an exploring and exploiting method tested in previous studies.^19^

This is supported by a study by Gorantla et al., who tested multiple setup configurations for four different targets. These setups used Chemprop or a Gaussian process regression model as ML algorithm; a random, uncertainty-based, greedy, or a mixed acquisition method; increasing initial batch sizes of 60 or 120 molecules for an exploration phase; and iterative batch sizes with 20, 30, 60, or 120 molecules per iteration for an exploitation phase.^20^ For more chemically diverse and bigger data sets, the recall number benefits from a bigger initial batch size. The bigger the initial batch size is, the higher are the recall numbers for all data sets, but experiments with smaller data sets could benefit from the trade-off of saving on computational costs with a smaller number of samples overall. For iterative batch sizes, smaller batch sizes resulted in higher recall numbers, indicating that these batch sizes favor precision and recall. Bigger training sets for the ML model in the exploitation phase could lead to noise and worsen predictive performance.

Gusev et al. suggest combining an exploring strategy in the first iterations with a narrowing (exploiting) technique afterward.^21^ After selecting a diverse initial sample set, five cycles with a balanced selection method, one cycle with a randomly selected sample set, and one cycle with an exploitive selection method were used. Gusev et al. used AutoML to select an ML model algorithm, hyperparameters, and data representation for every iteration to generate the best ML model configuration for each cycle.^26^ AutoML was presented with molecular descriptors PLEC, Morgan fingerprints, RDKit 3D molecular fingerprints, and RDKit molecular fingerprints; and ML algorithms linear regression, Gaussian process regression, and Gaussian Process regression with Tanimoto kernel.^23,25,26^ This resulted in the configuration of five different ML models for seven iterative cycles. This led to identifying 133 potential new inhibitors for severe acute respiratory syndrome coronavirus 2 papain-like protease while sampling 253 compounds out of a data set of 8175.^21^

Recently, a study by Lonsdale et al. presented a systematic benchmarking of AL-FEP for ligand potency prediction in lead optimization. A distinctive focus of their study is the role of chemical diversity, comparing AL-FEP performance on two bromodomain inhibitor series: one with a constant core scaffold and another allowing for core modifications. They demonstrate that while AL-FEP can efficiently generate predictive ML models within a few rounds, the effectiveness is highly context-dependent. With a low scaffold diversity, models rapidly achieve high accuracy and effectively enrich potent binders. However, pools with diverse scaffolds challenge the ML model’s performance, leading to reduced prediction quality for underrepresented scaffolds.^22^ Lonsdale et al. test a range of selection strategies, including explore–exploit mixture strategies, a grid-diversified selection method, and a greedy-diversified selection method. They show that protocol choice determines whether the model excels at finding top binders or provides broad accuracy across the potency space, especially when looking at diverse scaffolds. While a greedy selection strategy may be effective when maximizing binding potency alone, for optimizing multiple properties or exploring multiple end points, an exploratory model that maintains high-potency predictive accuracy across multiple scaffolds is preferable.^22^

Collectively, these five studies underline the potential of AL strategies to enhance the efficiency of FEP screening, with reported speed improvements of up to 20-fold compared to conventional brute-force methods.^19^ The effectiveness of AL can be further optimized by adjusting various parameters, such as acquisition strategies, batch sizes, and initial training sets, based on the specific objectives of a given experiment. Caution however is warranted, as the optimal combination of these parameters is target and data set dependent^20^, making it challenging to draw general conclusions. Efforts to build community benchmarks, such as those by Thompson et al., are needed to progress the field. However, even with the large (10,000 ligands) data set reported in their work, they selected similar compounds to the reference compound, which may reduce the predictive accuracy of AL-FEP methods when trying to find chemical diverse new compounds.^19^ It is additionally worth mentioning that these five papers are retrospective studies, and whether the optimization strategies truly improve screening effectiveness remains to be studied prospectively.

### 2.2 FEP Protocol and Protein Setup

With recent advancements in the efficiency of FEP calculations, the primary bottleneck has shifted from computation to the meticulous setup required for accurate simulations. Each protein system necessitates a customized approach, and ligand data sets must be precisely configured to ensure reliable binding affinity predictions. Critical factors such as the protonation states of titrable residues (particularly histidine) and correct inclusion of relevant (buried) water molecules within the binding site can significantly affect the accuracy of FEP outcomes.^27,28^ These system-specific variables demand deep insight and understanding of the protein–ligand complex and careful experimental design. ML and AL protocols could streamline these processes with less human interaction and preparation steps. Scheme 2a illustrates the current process used to prepare the FEP calculations. Scheme 2b provides a graphical overview of the preparation steps using recently developed modeling (ML) tools. In this section, we will discuss these tools in more detail.

**Scheme 2:**
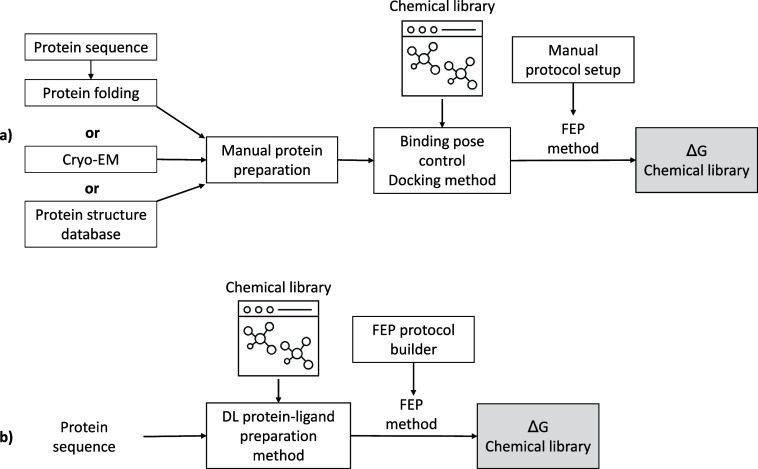
Comparison of the Schematic Workflow of Protein Setup for FEP Methods^a^

a (A) The traditional workflow includes protein structure acquisition, preparation, and binding pose creation before FEP. (B) An alternative workflow using cofolding techniques.

To streamline the process of FEP protocol optimization, de Oliveira et al. developed the FEP Protocol Builder (FEP-PB), an AL protocol that automates the creation of FEP+ protocols. This protocol enables optimization across multiple aspects of the FEP setup, including protein structure selection, custom core definition, equilibration time scale, water model and sampling strategies, lambda scheme, replica exchange settings for both protein and ligand, enhanced sampling settings for ligands, reference ligand selection, force field choice, and the tautomeric, protonation, and orientation states of residues. First, it generates random protocols for a series of compounds from a training set, which are subjected to a short 1 ns FEP simulation. The performance is assessed using the RMSE of the FEP predicted and experimental binding free energies, and an AutoML model^29^ is trained to predict and select top protocols, iteratively refining them with extended simulations for convergence before testing final protocols on the test set to identify the best-performing and robust model.^29^ In some cases, the optimized model outperformed protocols designed by experienced computational chemists in both speed and predictive accuracy.^30^

Cofolding methods are emerging as promising alternatives to traditional/AI docking techniques. These approaches predict protein–ligand complexes directly through joint folding of the ligand and protein, potentially yielding more accurate representations of binding poses, protein conformations, and local protonation states. Notable advancements in this area include AlphaFold3 by DeepMind and HelixFold3 by Furui and Ohue, both reporting improved accuracy in predicting protein–ligand complexes compared to conventional docking approaches.^4,31^ NeuralPLexer3 leverages a multiscale deep learning architecture to predict protein–ligand complex structures with a reported improvement in capturing ligand-induced conformational changes compared to AlphaFold3 and improves computational efficiency by being up to 15 times faster.^32^ In parallel, several open-source alternatives to AlphaFold3 have been developed, including Chai-1, Boltz-1, and Boltz-1x.^32–34^ While Boltz-1 primarily focuses on single-chain protein structure prediction in comparison to AlphaFold2, Chai-1 and Boltz-1x expand functionality toward multimolecular complex prediction using multiple sequence alignments (MSAs), chemical inputs, or even a single sequence.

Boitreaud et al. report that Chai-1 attains a performance comparable to AlphaFold3 across a range of targets, although its accuracy diminishes when wild-type residues are substituted with modified amino acids, a limitation likely rooted in its training on explicitly optimized structures and sequences. Additionally, Chai-1 performs well at predicting individual molecular structures but struggles with resolving correct relative orientations within complexes.^34^ In contrast, Boltz-1 and Boltz-1x exhibit comparable structural prediction performance and demonstrate superior physical plausibility, as measured by physical validity scores.^35^

The latest advancement in this family, Boltz-2, extends beyond structural prediction by incorporating a binding affinity estimation into its modeling framework. This dual capability enables Boltz-2 to generate both high-quality protein–ligand complex structures and approximate free energy predictions in a single step. Such integration is particularly promising for use in AL-FEP workflows, where rapid prescreening and prioritization of candidate compounds are essential. By supplying both structural models and binding affinity estimates, Boltz-2 offers an efficient means of selecting the most informative compounds for full FEP simulations, thereby reducing the computational cost while maintaining predictive accuracy.

Charm Therapeutics combined cofolding with FEP using an algorithm named DragonFold.^36^ This tool aims to skip traditional docking procedures by predicting protein–ligand structures, increasing the accuracy of the protein structure and the binding position of the ligand in the binding pocket, while skipping a time-consuming preparation step. In addition, truncated representations of the system are introduced, similar to other methods such as MCPRO and QligFEP.^37,38^ . A study on PFKFB3 showed that similar performance on the cofolded system could be achieved compared to the X-ray and docking starting structures. In addition, it was shown that the performance on the truncated systems was equivalent (∼1 kcal/mol MUE on average) to the full systems but with an overall increase in performance of 2- to 4-fold.

Third-party benchmarking studies have recently provided further insights into the capabilities and limitations of cofolding and AI methods in protein–ligand complex prediction. Large-scale evaluations such as PoseX and Runs N’ Poses have compared AI cofolding approaches, including AlphaFold3, Chai-1, Boltz-1/1x, Protenix, and NeuralPLexer3, against both traditional docking and each other using independent data sets released after the methods’ training cutoffs. Collectively, these studies reveal that while cofolding models outperform classical docking in pose prediction and structural plausibility in favorable scenarios, their success remains strongly tied to the similarity of the test system to training data, raising concerns about overfitting and generalization to novel targets.^39,40^ The Runs N’ Poses benchmark demonstrates that cofolding models can struggle to correctly predict ligand binding modes for drug-like molecules not well-represented in their training sets, although they perform better with promiscuous ligands or cofactors encountered more frequently in experimental structures.^40^ Meanwhile, physical validity analyses confirm that recent advances (e.g., Boltz-1x) lead to improved stereochemistry and plausibility of generated complexes compared to prior iterations^39,41^ Crucially, benchmarking also highlights persistent issues such as handling of chirality, ranking of alternative poses, and reduced accuracy when significant conformational rearrangements or post-translational modifications are present. These findings underline both the promise and the challenges of cofolding.

### 2.3 Force Field Enhancements

MD simulations and FEP calculations are critically dependent on the accuracy and reliability of the underlying force fields (FFs). Current FEP workflows predominantly rely on molecular mechanics (MM) force fields, which use physics-based models to simulate interatomic interactions such as bond stretching, angle bending, torsional rotation, and nonbonded forces. A variety of both open-source and commercial MM force fields have been developed, many of which offer comparable levels of accuracy across a broad range of chemical systems.^42–46^ Despite ongoing improvements, limitations in the transferability and parametrization of MM force fields remain a source of uncertainty in binding free energy predictions, highlighting the need for continued refinement and benchmarking across diverse molecular contexts.

While molecular mechanics (MM) force fields offer the efficiency required for large-scale simulations, they are limited in their ability to capture complex electronic effects. In contrast, more accurate force fields can be derived from quantum mechanical (QM) or hybrid QM/MM methods, which directly model electronic interactions. However, the computational cost of these methods makes them impractical for routine use in FEP workflows. As a result, QM calculations are typically restricted to small, chemically diverse molecules and used in conjunction with MM force fields to improve the accuracy in specific contexts, for example, the use of density functional theory calculations done by Bui et al. for geometry optimization of potential drug molecules.^47^

To bridge the gap between accuracy and efficiency, machine-learning-derived force fields, commonly termed neural network potentials (NNPs), have been developed. These models are trained on high-level QM data and aim to replicate QM-level accuracy with significantly reduced computational cost.^48–54^ Scheme 3 shows a graphical overview of the generation of MM (3a) and QM/MM or NNP (3b) force fields. Recent efforts have focused on enhancing the transferability of NNPs across different molecular states, as demonstrated by Kovács et al. in the development of MACE-OFF.^55^ In addition, attention has been directed toward improving data efficiency through innovative training strategies and increasing robustness against noisy input data.^48–55^

**Scheme 3:**
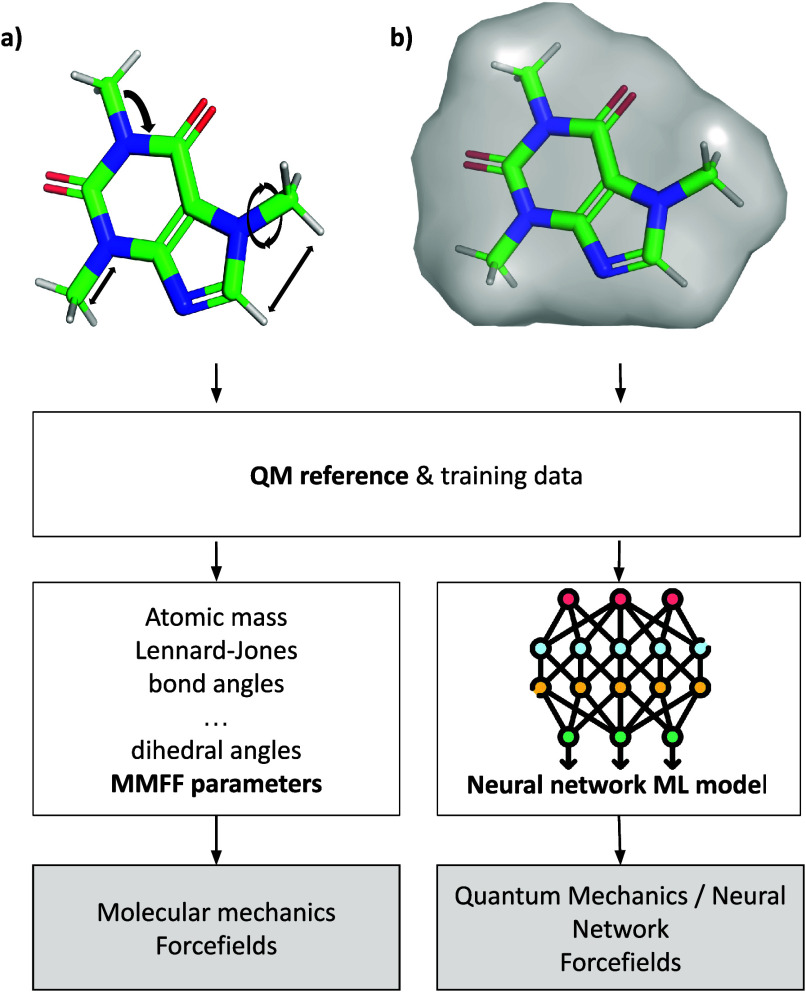
Schematic Workflow for Obtaining (A) MM Force Fields and (B) QM/NN Force Fields

Despite encouraging results, several challenges remain in the practical integration of the NNP and MM/NNP hybrid force fields. For instance, widely used models such as ANI are currently limited in scope to small molecules.^48,49,56^ These methods typically remain significantly slower and more resource-intensive than conventional MM force fields, which limit their scalability in high-throughput settings or larger systems such as protein systems. Continued advancements in elemental coverage, solvent modeling, and computational efficiency will be essential for the broader adoption of NNP-based force fields in FEP applications.

In this context, significant progress has been made by Anstine et al. with the development of AIMNet2, a neural network potential designed to overcome several enduring limitations of previous machine learning potentials.^57^ AIMNet2 offers broad elemental coverage and supports both neutral and charged molecular states. Its architecture effectively integrates machine-learned short-range interactions with physics-based long-range terms such as explicit electrostatics and dispersion, enabling accurate representation of both local and nonlocal effects. Benchmark studies demonstrate that AIMNet2 achieves accuracy on par with hybrid density functional theory methods across a variety of molecular modeling challenges, including conformer generation, interaction energy calculations, geometry optimization, and molecular dynamics, while considerably outperforming traditional semiempirical approaches in both chemical space coverage and computational efficiency. Importantly, AIMNet2 models are made available as open-source tools and can be applied directly to diverse chemical systems without requiring retraining, representing a substantial advancement toward the integration of machine-learning-based force fields into scalable, automated molecular simulation workflows.^57^

Zariquiey et al. conducted a systematic evaluation of the ANI-2x NNP for RBFE applications, benchmarking its performance against the GAFF2 force field and the commercial FEP+ platform across a range of protein–ligand systems.^58^ Their analysis revealed that ANI-2x did not produce substantially different conformational ensembles compared to traditional force fields, with only modest improvements in predictive accuracy observed. However, these gains came at a significant computational cost, approximately 8-fold higher than FEP+ when run on an NVIDIA RTX 4090 GPU. While NNP force fields like ANI-2x offer the potential for enhanced accuracy in challenging systems, they currently lack the efficiency and scalability of established MM force fields. Nonetheless, they may be valuable in cases where MM force fields fail to yield reliable results, serving as an intermediate between classical and fully QM-based methods.

A comprehensive study by Karwounopoulos et al. benchmarked the ANI-2x neural network potential, applied both for ML/MM end-state corrections and for reparametrizing torsion potentials within MM force fields across several drug discovery benchmark systems. Their findings show that ML/MM methods achieve comparable accuracy to well-parametrized MM force fields, with average errors of around 0.8–0.9 kcal/mol. However, ML/MM end-state corrections exhibited higher variance and computational costs in comparison to the torsion refitting approach, which provides a computationally efficient alternative without sacrificing accuracy. The limited improvement from ML/MM mechanical embedding is attributed to the unchanged MM treatment of protein–ligand interactions, underscoring the need for enhanced phase-space overlap and more advanced embedding schemes in future developments. These insights emphasize that ML-driven torsion parameter refinement can be a practical strategy for improving force fields in automated drug discovery pipelines with minimal computational overhead.^59^

More recently, the same group introduced a hybrid NNP/MM approach, QuantumBind-RBFE, which was also benchmarked against GAFF2 and ANI-2x.^60^ Quantumbind-RBFE uses the NNP AceFF1.0, showing improved accuracy in binding free energy predictions and supporting larger integration timesteps (2 versus 1 fs) without compromising simulation stability on the same GPU hardware (AceFF1.0 model: https://huggingface.co/Acellera/AceFF-1.0). These enhancements mark progress toward more practical ML-augmented force fields for FEP workflows. However, AceFF1.0, like any ML model, is dependent on the diversity and representativeness of its training data.

To further address the computational challenges of NNPs, Tkaczyk et al. developed an alternative strategy that reweights MM force field simulations using ANI-2x.^61^ This method offers a more computationally efficient pathway than direct NNP-based dynamics while retaining the accuracy benefits associated with neural network potentials. When evaluated for single-step FEP and nonequilibrium (NEQ) free energy calculations, ANI-2x reweighting yielded mixed results: the performance in NEQ calculations was promising, but single-step FEP results were found to be less accurate. These findings highlight the ongoing trade-offs between speed, accuracy, and methodological robustness in efforts to integrate ML-derived potentials into FEP simulations. Continued development of hybrid workflows, expanded training sets, and adaptive integration strategies will be crucial for realizing the full potential of NNPs in drug discovery pipelines.

The OpenMM simulation environment has also integrated support for hybrid NNP/MM methodologies, in which neural network potentials are applied to small molecules while the surrounding environment is treated with conventional molecular mechanics force fields.^62^ Building on this capability, Ding et al. introduced a deep-learning-based potential model, known as OpenMM-DeepMD, which enables the use of high-precision machine learning force fields implemented in C++/CUDA and accessible through a Python interface.^63^ The DeepMD plugin was rigorously validated through a series of benchmarks, including energy conservation tests, thermodynamic ensemble consistency, structural and kinetic property evaluations, and hydration free energy calculations. In all cases, the results were consistent with the experimental reference data, confirming both the accuracy and robustness of the implementation.

Notably, the plugin supports both fixed- and adaptive-region deep potential/MM (DP/MM) simulations, allowing for dynamic partitioning of the system into machine learning and classical regions. Performance benchmarks demonstrated simulation speeds of up to 159 ns/day on an NVIDIA 1060 Ti GPU, showcasing the method’s practical efficiency. By integration of the deep potential model directly into OpenMM, this plugin enables accurate, flexible, and scalable ML-based molecular simulations, including advanced applications such as free energy calculations and hybrid modeling. This development represents a meaningful step toward the routine use of machine learning force fields in large-scale, physics-informed molecular simulations.

## 3 Conclusion and Perspectives

In recent years, significant progress has been made in enhancing FEP methodologies through the integration of ML techniques. This review has outlined key developments across several fronts, including sampling strategies, automated protocol generation, protein structure prediction, and cofolding models, as well as advancements in force field design. While improvements in sampling and protocol automation are primarily aimed at increasing the efficiency and scalability of FEP workflows, innovations in protein modeling and force field development target higher accuracy and broader applicability.

One of the most promising directions involves the use of ML-enhanced or NNP-hybrid force fields. Although these approaches are currently more computationally intensive than classical MM force fields, they have the potential to become standard practice as computational resources continue to advance. Importantly, the development of high-accuracy force fields extends beyond drug discovery, with significant implications for materials science, chemical engineering, and other domains that rely on molecular dynamics simulations. This cross-disciplinary relevance is likely to accelerate innovation, particularly if research findings are openly shared across academia, industry, and related scientific fields.

Cofolding models such as Boltz-1x and AlphaFold3 also hold considerable potential to simplify and democratize FEP workflows. The traditional protein setup for FEP requires deep expertise in structural biology and computational biochemistry, often making it inaccessible to nonspecialists. In contrast, cofolding models generate plausible protein–ligand complex structures using only the primary sequence and ligand input, significantly lowering the technical barrier. However, the current models are not without limitations. First, their performance drops with novel drug-like molecules not represented in training sets. Advances like Boltz-1x improve stereochemistry and physical validity, yet challenges remain in chirality handling, pose ranking, accuracy with major conformational changes, and modeling of wild-type substitutions. Second, all cofolding approaches still require greater computational resources compared to standard docking and protein preparation methods. Recent developments such as DragonFold and its truncated models represent an important step forward by offering lower computational demands while maintaining compatibility with FEP requirements.

Overall, cofolding shows promise but also clear limitations in generalizability and pose prediction reliability. In parallel, automated protocol-building tools such as the FEP Protocol Builder (FEP-PB) further reduce the user burden, enabling both novice and experienced users to generate high-quality FEP protocols. While fully automated systems have shown promising results, a hybrid human–ML approach may offer the optimal balance of efficiency, flexibility, and scientific rigor. This paradigm could extend to other aspects of the FEP pipeline, as well. For instance, in large-scale virtual screening campaigns, current ML models remain limited in their ability to assess synthesizability, off-target effects, and in vivo behavior. Integrating expert knowledge, particularly from medicinal chemists, into a “human-in-the-loop” framework could significantly enhance the downstream quality of candidate molecules.

Taken together, these developments suggest a future in which FEP workflows will be faster, more accurate, and more accessible. Continued investment in ML-guided automation, hybrid modeling strategies, and interdisciplinary collaboration will be essential to realize this vision and to extend the impact of FEP across a wider range of scientific and industrial applications.

## ABBREVIATIONS

CADD: computer-aided drug discovery
SBDD: structure-based drug design
MD: molecular dynamics
FEP: free energy perturbation
ML: machine learning
resFEP: residue free energy perturbation
ABFE: absolute binding free energy
RBFE: relative binding free energy
TI: thermodynamic integration
(M)­BAR: (multistate) Bennet acceptance ratio
KRAS: Kirsten rat sarcoma
TYK2: tyrosine kinase 2
PDE2A: phosphodiesterase 2A
c-MET: mesenchymal–epithelial transition factor
A2BAR: A2b adenosine receptor
DL: deep learning
AL: active learning
QSAR: quantitative structure–activity relationship
MLP: multilayer perceptron
FEP-PB: FEP Protocol Builder
MM: molecular mechanics
QM: quantum mechanics
NNP: neural network potential
FF: force field
ANI: Accurate NeurAl networK engine
MACE-OFF: MACE-based neural potential-Optimization for Force Field
MUE: mean unsigned error
NEQ: nonequilibrium
DP: deep potential
DP/MM: deep potential/molecular mechanics
GPU: graphics processing unit
PFKFB3: 6-phosphofructo-2-kinase/fructose-2,6-biphosphatase 3
X-ray: X-ray crystallography
MSA: multiple sequence alignment
OpenMM: Open Molecular Mechanics
